# Crystal structures of *p*-substituted derivatives of 2,6-di­methyl­bromo­benzene with ½ ≤ *Z*′ ≤ 4

**DOI:** 10.1107/S2056989016017485

**Published:** 2016-11-08

**Authors:** Angélica Navarrete Guitérrez, Gerardo Aguirre Hernández, Sylvain Bernès

**Affiliations:** a Centro de Graduados e Investigación en Química, Instituto Tecnológico de Tijuana, Apartado Postal 1166, 222000 Tijuana, B.C., Mexico; b Instituto de Física, Benemérita Universidad Autónoma de Puebla, Av. San Claudio y 18 Sur, 72570 Puebla, Pue., Mexico

**Keywords:** crystal structure, bromo­arenes, *Z*′, hydrogen bond, mol­ecular symmetry

## Abstract

The crystal structures of four bromo­arenes based on 2,6-di­methyl­bromo­benzene are reported, which are differentiated according the functional group *X* placed *para* to the Br atom: *X* = CN (4-bromo-3,5-di­methyl­benzo­nitrile, C_9_H_8_BrN), (**1**), *X* = NO_2_ (2-bromo-1,3-dimethyl-5-nitro­benzene, C_8_H_8_BrNO_2_), (**2**), *X* = NH_2_ (4-bromo-3,5-di­methyl­aniline, C_8_H_10_BrN), (**3**) and *X* = OH (4-bromo-3,5-di­methyl­phenol, C_8_H_9_BrO), (**4**). The content of the asymmetric unit is different in each crystal, *Z*′ = ½ (*X* = CN), *Z*′ = 1 (*X* = NO_2_), *Z*′ = 2 (*X* = NH_2_), and *Z*′ = 4 (*X* = OH), and is related to the mol­ecular symmetry and the propensity of *X* to be involved in hydrogen bonding. In none of the studied compounds does the crystal structure feature other non-covalent inter­actions, such as π–π, C—H⋯π or C—Br⋯Br contacts.

## Abstract 2

Four bromo­arenes are characterized by different contents for their asymmetric units, with *Z*′ varying from ½ to 4, depending on the nature of a single functional group.

## Chemical context   

Our group is inter­ested in the design of chemical model systems for studying polar–π inter­actions (Cozzi *et al.*, 2008 ▸). In order to achieve this objective, it is necessary to prepare a variety of aryl­boronic esters as suitable substrates for Suzuki–Miyaura cross-coupling reactions (Ishiyama *et al.*, 1995 ▸; Kotha *et al.*, 2002 ▸). We obtained these boronic derivatives starting from functionalized bromo­arenes. The present communication is about the synthesis and crystallography of a series of such bromo­arenes, namely, *para*-substituted derivatives of 2,6-di­methyl­bromo­benzene, for which the *p*-substituent is *X* = CN (**1**), *X* = NO_2_ (**2**), *X* = NH_2_ (**3**), or *X* = OH (**4**).
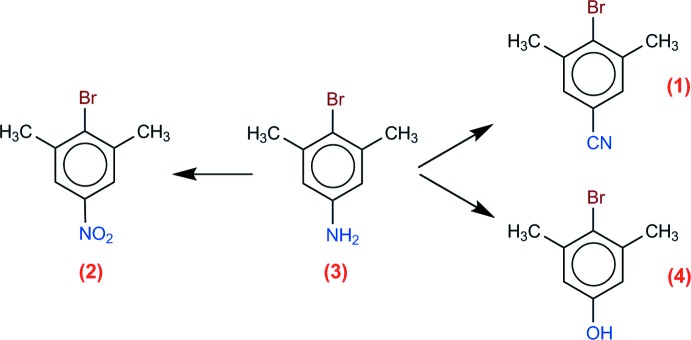


The crystallized mol­ecules are closely related to one another from the chemical and structural points of view. However, very different crystal structures were obtained, with different compositions for the asymmetric units. Once again, this evidences that small chemical modifications for a given compound may induce dramatic changes in its crystal structure, even in the case of hydrogen/deuterium exchange, which is the smallest possible modification of a mol­ecule (Vasylyeva *et al.*, 2010 ▸). As a consequence, the blind tests of organic crystal-structure prediction hosted by the CCDC (Reilly *et al.*, 2016 ▸) certainly have a bright future ahead of them.

## Structural commentary   

**Figure 1:**
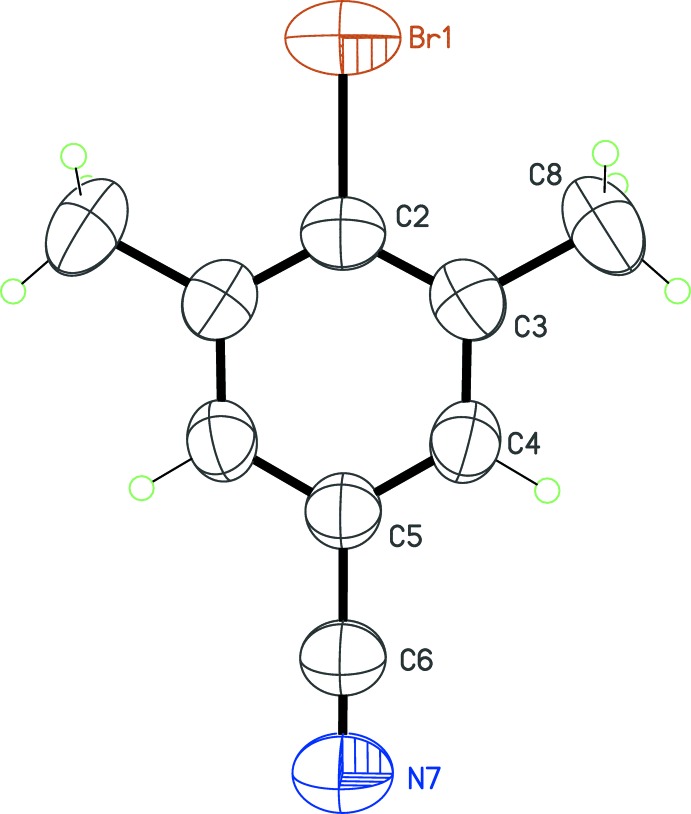
The mol­ecular structure of (**1**), with displacement ellipsoids for non-H atoms at the 50% probability level. Unlabelled atoms are generated by the symmetry operation (*x*, 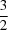 − *y*, *z*).

No unusual bond lengths or angles are observed in the four mol­ecules (Figs. 1 ▸–4 ▸ ▸ ▸). For example, the C—Br bond lengths span a narrow range, from 1.900 (4) to 1.910 (2) Å. The substituent *X* in the position *para* to the C—Br bond thus has no influence on the geometry of the bromo­benzene core, even if very different *X* groups are used, namely, strongly electron-withdrawing groups (*X* = CN, NO_2_) and strongly electron-donating groups (*X* = NH_2_, OH). Another structural invariant over the studied series is the minimization of steric crowding effects between the Br atom and the methyl groups in *ortho* positions. The methyl groups are systematically rotated in such a way that the C—Br bond is staggered with a CH_2_ fragment of the methyl group. As a consequence, the endocyclic angle at the Br-bearing C atom is always the largest one in the benzene ring, varying from 121.8 (3)° in (**3**) to 123.9 (4)° in (**1**).

**Figure 2:**
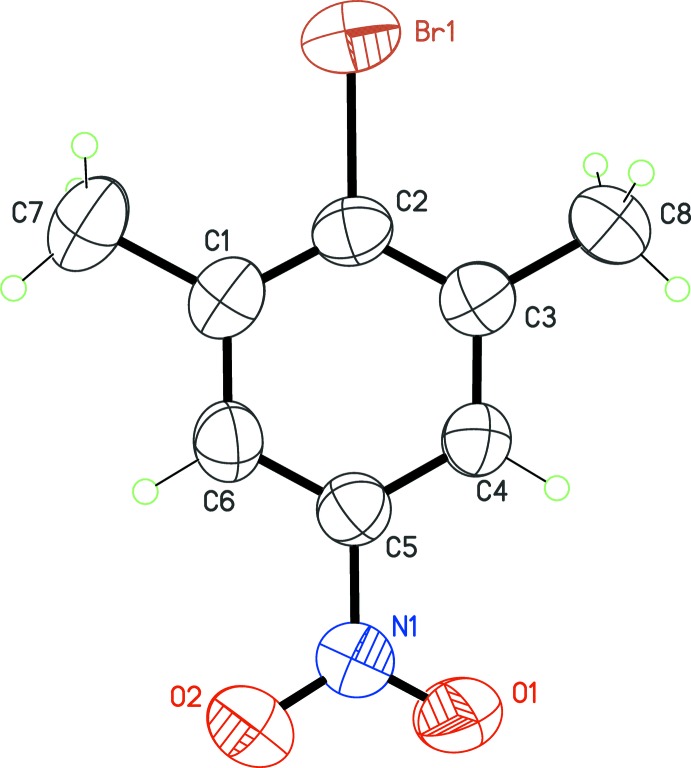
The mol­ecular structure of (**2**), with displacement ellipsoids for non-H atoms at the 50% probability level.

**Figure 3:**
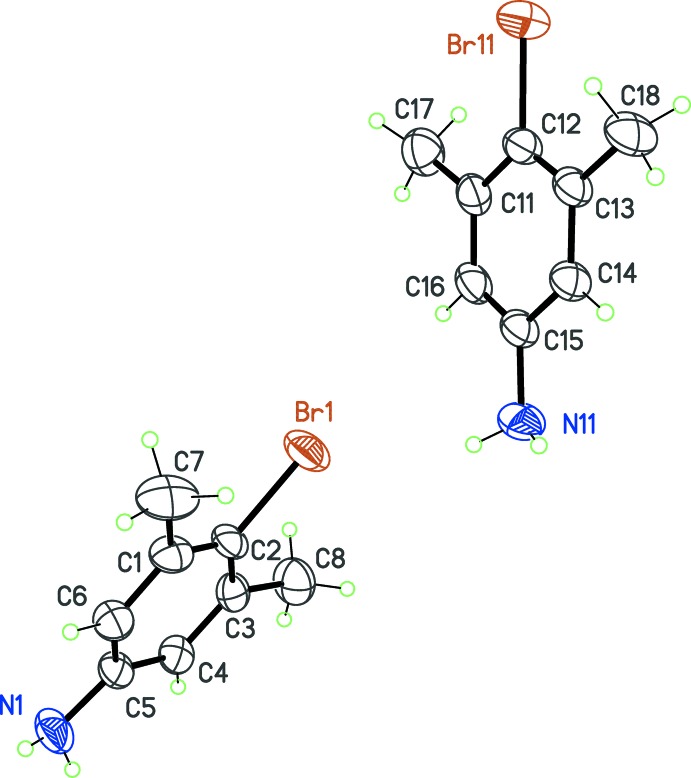
The asymmetric unit of compound (**3**), with displacement ellipsoids for non-H atoms at the 30% probability level.

**Figure 4:**
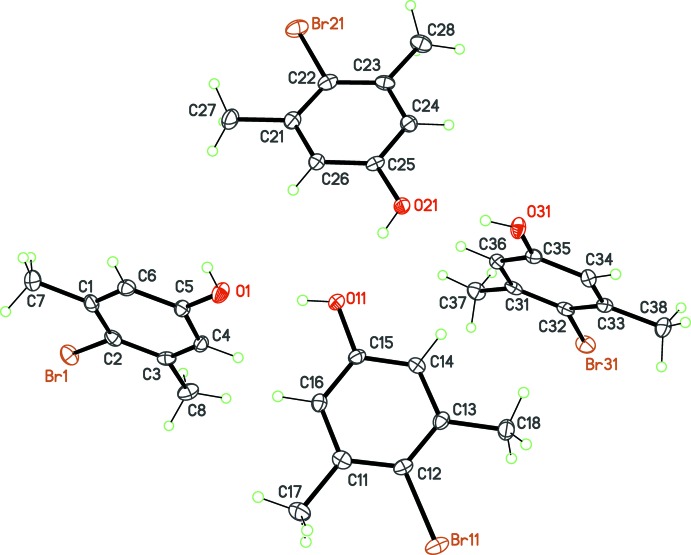
The asymmetric unit of compound (**4**), with displacement ellipsoids for non-H atoms at the 50% probability level.

The point of inter­est regarding the mol­ecular structures is that four different values of *Z*′ are obtained for the four compounds. Mol­ecule (**1**) (*X* = CN) has the highest potential mol­ecular symmetry, *C*_2*v*_, assuming a linear C—C≡N group. Omitting H atoms, this symmetry is actually reached, with the C—Br and C—C≡N fragments lying on the mirror plane in space group *P*2_1_/*m* (Fig. 1 ▸). The asymmetric unit then contains a half-mol­ecule, and *Z*′ = ½. In (**2**), with *X* = NO_2_, the latent symmetry *C*_2*v*_ is broken because the nitro group is tilted slightly with respect to the benzene ring by an angle of 13.0 (4)°. For this crystal, *Z*′ = 1 in space group *P* 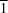 (Fig. 2 ▸). Finally, for (**3**) and (**4**), which are isoelectronic mol­ecules [*X* = NH_2_, (**3**) and *X* = OH, (**4**)], despite the mol­ecular symmetry being close to *C*_2*v*_, the asymmetric units contain more than one mol­ecule: *Z*′ = 2 for (**3**) (Fig. 3 ▸) and *Z*′ = 4 for (**4**) (Fig. 4 ▸), in space groups *P*2_1_/*n* and *Pbca*, respectively.

The increasing size of the asymmetric unit, reflected in the increasing value of *Z*′, may be rationalized on the basis of two key parameters. First, a higher mol­ecular symmetry obviously favours the crystallization of low *Z*′ crystals, as in (**1**). This has been observed in many symmetrically substituted benzene derivatives, for example, in 4-bromo-benzo­nitrile in space group *Cm* (Britton *et al.*, 1977 ▸; see also Desiraju & Harlow, 1989 ▸), or 2,6-di­bromo-4-chloro­benzo­nitrile in space group *P*2_1_/*m* (Britton, 2005 ▸). The standard asymmetric unit with *Z*′ = 1 is obtained for (**2**), for which the mol­ecular symmetry is lowered to *C*_1_. Secondly, the introduction of efficient donor groups for hydrogen bonding, such as NH_2_ and OH groups, is an enabling factor for crystal structures having *Z*′ > 1, as observed for (**3**) and (**4**). A search in the organic subset of the CSD (Groom *et al.*, 2016 ▸) reflects such a trend: for example, comparing nitro­benzene and aniline derivatives, the former class is characterized by 12.5% of crystals with *Z*′ > 1, and this fraction is increased to 15.6% in the latter. In the same way, phenol derivatives with *Z*′ = 4 are not uncommon (Dey *et al.*, 2005 ▸; Mukherjee & Desiraju, 2011 ▸).

## Supra­molecular features   

As expected, compound (**1**) is featureless regarding the packing of the mol­ecules. No short contacts such as halogen bonds are formed, and π–π inter­actions are insignificant, the shortest separation between benzene ring being defined by cell translations along the short cell axis, *a* = 4.0382 (1) Å.

**Figure 5:**
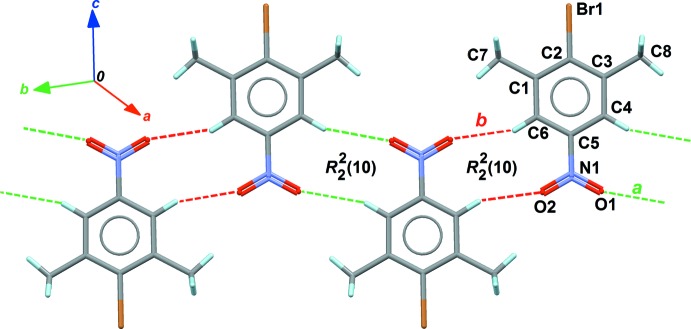
Part of the crystal structure of (**2**), showing C—H⋯O hydrogen bonds (dashed lines) forming *R* motifs in the crystals. Hydrogen bonds *a* (green) and *b* (red) correspond to entries 1 and 2 in Table 1 ▸. Atoms belonging to the asymmetric unit are labelled.

For (**2**), two pairs of weak C—H⋯O hydrogen bonds link the mol­ecules to form two centrosymmetric first-level ring motifs of *R^2^*_2_(10), with the participation of the nitro group as acceptor (Table 1 ▸). The nitro group participates with two contacts to two rings, generating a chain of *R* motifs along [1
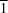
0] (Fig. 5 ▸). As for (**1**), slipped π-stacking inter­actions are insignificant, the benzene-to-benzene distance being, again, determined by the cell axis *a* = 4.0502 (5) Å.

Although compounds (**3**) and (**4**) are isoelectronic, they present different crystal structures. This is because their donor groups for hydrogen bonding are of a different nature: the N—H bond is a poorer donor compared to the O—H bond, on the basis of the polarity of these bonds, estimated with the differences of electronegativity χ_N_ − χ_H_ = 0.84 and χ_O_ − χ_H_ = 1.24 (Pauling’s scale is used for χ). Moreover, the NH_2_ group is potentially involved in two hydrogen bonds, while the OH group is expected to form a single, stronger contact, at least as long as bifurcated hydrogen bonds are not considered.

**Figure 6:**
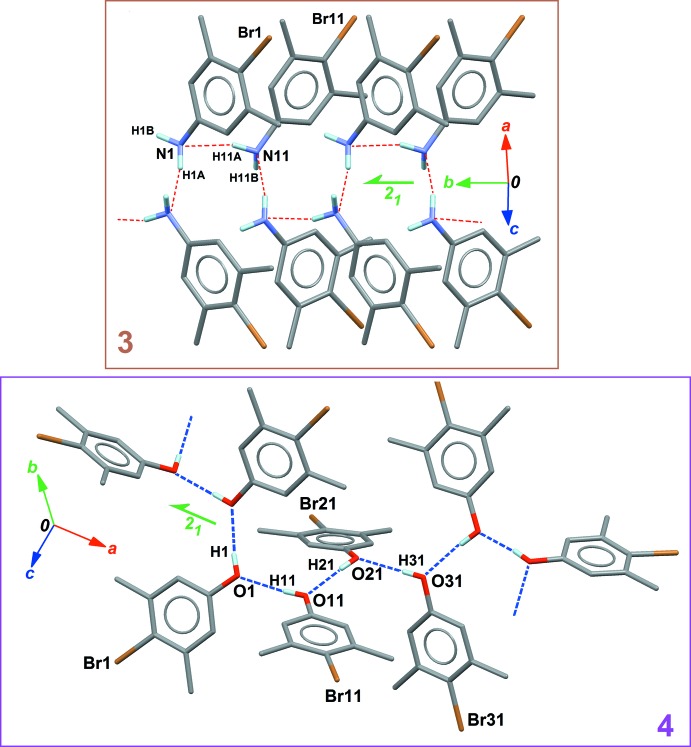
Part of the crystal structures of (**3**) (top) and (**4**) (bottom), showing N—H⋯N and O—H⋯O hydrogen bonds (red and blue dashed lines, respectively). In each case, the asymmetric unit comprises the mol­ecules with labelled atoms.

Both compounds (**3**) and (**4**) have a supra­molecular structure based on chains oriented along a screw 2_1_ axis (Fig. 6 ▸). For (**3**), two discrete contacts *D*(2) are formed between the two independent mol­ecules (Table 2 ▸). These contacts involve only one N—H bond for a given NH_*2*_ group, and the acceptor atom is the N site of the connected mol­ecule, with the N—H⋯N contact oriented toward the lone pair of the acceptor N atom. A second level motif 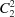
(4) is formed using the discrete contacts, and the chain of connected mol­ecules runs along [010] (Fig. 6 ▸, top).

A similar framework of *D* and *C* motifs appears in (**4**), this time starting from a *Z*′ = 4 asymmetric unit: three discrete motifs *D*(2) are formed within the asymmetric unit, and a fourth *D*(2) motif connects the first independent mol­ecule with a symmetry-related mol­ecule in the crystal (Table 3 ▸). As a consequence, 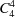
(8) chains are formed, propagating parallel to [100] (Fig. 6 ▸, bottom). As mentioned above, the hydrogen bonds in (**4**) are much more efficient than those observed in (**3**): all O—H⋯O bonds have short H⋯O distances of *ca* 1.9 Å and O—H⋯O angles are close to 180° (Table 3 ▸).

It is worth noting that none of the observed 1D supra­molecular structures in (**2**)–(**4**) include π–π or C—H⋯π contacts, nor C—Br⋯Br halogen bonds. The arrangement of the mol­ecules in the crystal over the studied series of compounds is thus mainly determined by the absence of, the presence of weak, or strong hydrogen bonds, respectively, in (**1**), (**2**) and (**3**), or (**4**).

## Database survey   

Polysubstituted benzene systems are ubiquitous in the crystallographic literature. Limiting a survey to 2,6-di­methyl­bromo­benzene, only two derivatives closely related to the series we have studied may be found, with *X* = ^*t*^Bu (Field *et al.*, 2003 ▸) and *X* = I (Liu *et al.*, 2008 ▸), which do not present obvious supra­molecular features. Both form *Z*′ = ½ crystals, as for (**1**).

## Synthesis and crystallization   

Compound (**3**) was purchased from Oakwood Chemical Co. and was the starting material for the synthesis of (**2**) by oxidation with *m*-CPBA, and (**1**) and (**4**) *via* a Sandmeyer reaction. Single crystals of (**3**) were obtained by slow evaporation of a CH_2_Cl_2_ solution.

Compound (1) was prepared by modification of the reported procedure (Xu *et al.*, 2000 ▸). A solution of NaNO_2_ (0.36 g, 5.2 mmol) in water (5 ml) was added dropwise to a suspension of 4-bromo-3,5-di­methyl­aniline (1 g, 5 mmol) in aqueous HCl (2 ml, 12 M), and water (2 ml) at 273 K. The mixture was stirred at 273 K for 30 min and then neutralized with NaHCO_3_. Separately, a solution of CuCN (0.54 g, 6 mmol), and KCN (0.81 g, 12 mmol) in water (10 ml) was heated at 343 K. This solution was added dropwise to the diazo­tization solution previously prepared. The mixture was kept at 343 K for 30 min with stirring and then cooled at room temperature. The product was extracted with toluene (3 × 30 ml). The combined organic layers were dried over anh. Na_2_SO_4_ and concentrated under reduced pressure. The crude product was purified by silica gel column chromatography (petroleum ether/EtOAc, 95:5) to obtain compound (**1**) as orange needles (0.77 g, 73%); m.p. 408–410 K; IR: 3022 (C—H Ar), 2354 (C≡N), 1498 (C=C) cm^−1^; ^1^H NMR (400 MHz, CDCl_3_): δ 7.34 (*s*, 2H), 2.44 (*s*, 6H) p.p.m.; ^13^C NMR (100 MHz, CDCl_3_) δ: 140.0, 133.2, 131.1, 118.4, 110.7, 23.8 p.p.m.; GC–MS (EI): *m*/*z* = 209 (100%) [*M* ^+^], 211 (97%) [*M* ^+^ + 2] amu. Single crystals suitable for X-ray analysis were obtained by slow evaporation of a CH_2_Cl_2_ solution.

Compound (**2**) was prepared by modification of the reported procedure (Gilbert & Borden, 1979 ▸). A solution of 4-bromo-3,5-di­methyl­aniline and 3-chloro­per­oxy­benzoic acid (4 g, 23 mmol) in CH_2_Cl_2_ (35 ml) was heated at 323 K for 2 h. After cooling at room temperature, the precipitate was filtered off and the liquid phase was washed with NaOH (1 *M*, 3 × 50 ml). The organic layer was dried over anh. Na_2_SO_4_ and concentrated under reduced pressure. The residue was dissolved in glacial acetic acid (10 ml), and a solution of H_2_O_2_ (5 ml, 33% aq. solution) and glacial acetic acid (5 ml) was added at room temperature. Then, conc. HNO_3_ (0.5 ml) was slowly added and the mixture was heated to 363 K for 4 h. After cooling, the crude was treated with water (50 ml), and was extracted with CH_2_Cl_2_ (3 × 50 ml). The combined organic layers were dried over anh. Na_2_SO_4_ and concentrated under reduced pressure. The crude was purified on a silica gel column chromatography (petroleum ether) to give compound (**2**) as bright-yellow crystals (0.51 g, 44%); m.p. 478–483 K; IR: 2988 (C—H Aliph), 1558, 1340 (N—O) cm^−1^; ^1^H NMR (400 MHz, CDCl_3_): δ 7.92 (*s*, 2H), 2.51 (*s*, 6H) p.p.m.; ^13^C NMR (100 MHz, CDCl_3_): δ 146.3, 140.1, 134.8, 122.5, 24.1 p.p.m.; GC–MS (EI): *m*/*z* = 229 (100%) [*M* ^+^], 231 (97%) [*M* ^+^+2] amu. Crystals suitable for single crystal X-ray diffraction were obtained by slow evaporation of an ether solution.

Preparation of (**4**): A solution of 4-bromo-3,5-di­methyl­aniline (1 g, 5 mmol) in conc. H_2_SO_4_ (25 ml) and water (5 ml) was cooled to 273 K. Then a solution of NaNO_2_ (0.35 g, 5 mmol) in water (10 ml) was added dropwise under stirring. After additional 30 min the solution was refluxed for 30 min. The mixture was cooled and extracted with EtOAc (3 × 50 ml). The combined organic phases were dried over anh. Na_2_SO_4_ and concentrated under reduced pressure. The crude was purified by silica gel column chromatography (petroleum ether/EtOAc, 9:1) to provide the product (**4**) as pale-orange crystals (0.56 g, 55%); m.p. 386–388 K; IR: 3620 (O—H), 2987 (C—H aliph), 1590 (C=C Ar), 1120 (C—O) cm^−1^; ^1^H NMR (400 MHz, CDCl_3_): δ 6.57 (*s*, 2H), 4.99 (*s*, 1H), 2.34 (*s*, 6H) p.p.m.; ^13^C NMR (100 MHz, CDCl_3_): δ 153.9, 139.5, 118.3, 115.2, 23.8 p.p.m.; GC–MS (EI): *m*/*z* = 200 (100%) [*M* ^+^], 202 (97%) [*M* ^+^ + 2] amu. Crystals suitable for diffraction were obtained by slow evaporation of an EtOAc solution.

## Refinement   

Crystal data, data collection and structure refinement details are summarized in Table 4 ▸. At room temperature, compound (**3**) decomposes after a few minutes under Mo *K*α irradiation, but is stable for hours under Cu *K*α irradiation. For compound (**3**), H atoms of NH_2_ groups were located in a difference Fourier map and were refined with restraints of N—H = 0.89 (2) Å and H⋯H = 1.52 (2) Å. For (**4**), H atoms of OH groups were found in a difference map and refined freely. All other H atoms in (**1**)–(**4**) were refined as riding.

## Supplementary Material

Crystal structure: contains datablock(s) 1, 2, 3, 4, global. DOI: 10.1107/S2056989016017485/is5462sup1.cif

Structure factors: contains datablock(s) 1. DOI: 10.1107/S2056989016017485/is54621sup2.hkl

Structure factors: contains datablock(s) 2. DOI: 10.1107/S2056989016017485/is54622sup3.hkl

Structure factors: contains datablock(s) 3. DOI: 10.1107/S2056989016017485/is54623sup4.hkl

Structure factors: contains datablock(s) 4. DOI: 10.1107/S2056989016017485/is54624sup5.hkl

Supporting information file. DOI: 10.1107/S2056989016017485/is54621sup6.cml

Supporting information file. DOI: 10.1107/S2056989016017485/is54622sup7.cml

Supporting information file. DOI: 10.1107/S2056989016017485/is54623sup8.cml

Supporting information file. DOI: 10.1107/S2056989016017485/is54624sup9.cml

CCDC references: 1513710, 1513709, 1513708, 1513707

Additional supporting information:  crystallographic information; 3D view; checkCIF report

## Figures and Tables

**Table 1:** Hydrogen-bond geometry (Å, °) for (**2**)

*D*—H⋯*A*	*D*—H	H⋯*A*	*D*⋯*A*	*D*—H⋯*A*
C4—H4*A*⋯O1^i^	0.93	2.51	3.377 (5)	156
C6—H6*A*⋯O2^ii^	0.93	2.55	3.351 (5)	144

Symmetry codes: (i) 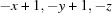; (ii) 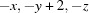.

**Table 2:** Hydrogen-bond geometry (Å, °) for (**3**)

*D*—H⋯*A*	*D*—H	H⋯*A*	*D*⋯*A*	*D*—H⋯*A*
N1—H1*A*⋯N11^i^	0.88 (2)	2.41 (3)	3.212 (6)	152 (5)
N11—H11*A*⋯N1^ii^	0.90 (2)	2.52 (3)	3.365 (6)	157 (4)

Symmetry codes: (i) 
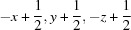
; (ii) 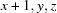.

**Table 3:** Hydrogen-bond geometry (Å, °) for (**4**)

*D*—H⋯*A*	*D*—H	H⋯*A*	*D*⋯*A*	*D*—H⋯*A*
O11—H11⋯O1	0.78 (3)	1.90 (3)	2.681 (3)	173 (4)
O21—H21⋯O11	0.76 (3)	1.92 (3)	2.682 (3)	176 (3)
O31—H31⋯O21	0.77 (3)	1.95 (3)	2.714 (2)	175 (3)
O1—H1⋯O31^i^	0.78 (3)	1.95 (3)	2.729 (3)	172 (3)

Symmetry code: (i) 
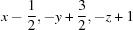
.

**Table 4:** Experimental details

	(**1**)	(**2**)	(**3**)	(**4**)
Crystal data				
Chemical formula	C_9_H_8_BrN	C_8_H_8_BrNO_2_	C_8_H_10_BrN	C_8_H_9_BrO
*M*_r_	210.07	230.06	200.08	201.06
Crystal system, space group	Monoclinic, *P*2_1_/*m*	Triclinic, *P* 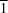	Monoclinic, *P*2_1_/*n*	Orthorhombic, *P* *b* *c* *a*
Temperature (K)	296	296	296	100
*a*, *b*, *c* (Å)	4.0382 (1), 8.9362 (4), 12.1015 (4)	4.0502 (5), 9.3817 (6), 12.1823 (5)	10.48314 (15), 6.10173 (10), 26.6195 (5)	14.65213 (17), 17.9520 (2), 24.0079 (3)
α, β, γ (°)	90, 93.763 (3), 90	93.498 (4), 99.284 (4), 101.722 (5)	90, 100.0731 (16), 90	90, 90, 90
*V* (Å^3^)	435.76 (3)	445.20 (7)	1676.48 (5)	6314.94 (12)
*Z*	2	2	8	32
Radiation type	Cu *K*α	Cu *K*α	Cu *K*α	Cu *K*α
μ (mm^−1^)	5.87	5.98	6.06	6.50
Crystal size (mm)	0.21 × 0.15 × 0.12	0.80 × 0.60 × 0.10	0.30 × 0.12 × 0.10	0.23 × 0.20 × 0.18

Data collection				
Diffractometer	Rigaku OD SuperNova AtlasS2	Rigaku OD SuperNova AtlasS2	Rigaku OD SuperNova AtlasS2	Rigaku OD SuperNova AtlasS2
Absorption correction	Multi-scan (*CrysAlis PRO*; Rigaku OD, 2015 ▸)	Multi-scan (*CrysAlis PRO*; Rigaku OD, 2015 ▸)	Multi-scan (*CrysAlis PRO*; Rigaku OD, 2015 ▸)	Multi-scan (*CrysAlis PRO*; Rigaku OD, 2015 ▸)
*T*_min_, *T*_max_	0.615, 1.000	0.304, 1.000	0.593, 1.000	0.601, 1.000
No. of measured, independent and observed [*I* > 2σ(*I*)] reflections	2457, 883, 771	5640, 1697, 1503	39830, 3276, 2716	22136, 6110, 5342
*R*_int_	0.027	0.038	0.093	0.033
(sin θ/λ)_max_ (Å^−1^)	0.615	0.616	0.620	0.615

Refinement				
*R*[*F* ^2^ > 2σ(*F* ^2^)], *wR*(*F* ^2^), *S*	0.044, 0.147, 1.08	0.045, 0.128, 1.07	0.054, 0.165, 1.11	0.026, 0.065, 1.02
No. of reflections	883	1697	3276	6110
No. of parameters	59	111	197	381
No. of restraints	0	0	6	0
H-atom treatment	H-atom parameters constrained	H-atom parameters constrained	H atoms treated by a mixture of independent and constrained refinement	H atoms treated by a mixture of independent and constrained refinement
Δρ_max_, Δρ_min_ (e Å^−3^)	0.51, −0.46	0.63, −0.59	0.44, −1.11	0.56, −0.43

Computer programs: *CrysAlis PRO* (Rigaku OD, 2015 ▸), *SHELXT* (Sheldrick, 2015*a* ▸), *SHELXL2014* (Sheldrick, 2015*b* ▸), *XP* in *SHELXTL* (Sheldrick, 2008 ▸), *Mercury* (Macrae *et al.*, 2008 ▸) and *CifTab* (Sheldrick, 2008 ▸).

## supplementary crystallographic information

### (1) Crystal data

**Table:**

C_9_H_8_BrN	*D*_x_ = 1.601 Mg m^−^^3^
*M**_r_* = 210.07	Melting point: 408 K
Monoclinic, *P*2_1_/*m*	Cu *K*α radiation, λ = 1.54184 Å
*a* = 4.0382 (1) Å	Cell parameters from 1415 reflections
*b* = 8.9362 (4) Å	θ = 3.7–71.2°
*c* = 12.1015 (4) Å	µ = 5.87 mm^−^^1^
β = 93.763 (3)°	*T* = 296 K
*V* = 435.76 (3) Å^3^	Needle, orange
*Z* = 2	0.21 × 0.15 × 0.12 mm
*F*(000) = 208	

### (1) Data collection

**Table:**

Rigaku OD SuperNova AtlasS2 diffractometer	883 independent reflections
Radiation source: micro-focus sealed X-ray tube, SuperNova (Cu) X-ray Source	771 reflections with *I* > 2σ(*I*)
Mirror monochromator	*R*_int_ = 0.027
Detector resolution: 5.1980 pixels mm^-1^	θ_max_ = 71.4°, θ_min_ = 3.7°
φ and ω scans	*h* = −3→4
Absorption correction: multi-scan (CrysAlis PRO; Rigaku OD, 2015)	*k* = −10→10
*T*_min_ = 0.615, *T*_max_ = 1.000	*l* = −14→13
2457 measured reflections	

### (1) Refinement

**Table:**

Refinement on *F*^2^	Primary atom site location: structure-invariant direct methods
Least-squares matrix: full	Secondary atom site location: difference Fourier map
*R*[*F*^2^ > 2σ(*F*^2^)] = 0.044	Hydrogen site location: inferred from neighbouring sites
*wR*(*F*^2^) = 0.147	H-atom parameters constrained
*S* = 1.08	*w* = 1/[σ^2^(*F*_o_^2^) + (0.0947*P*)^2^ + 0.0077*P*] where *P* = (*F*_o_^2^ + 2*F*_c_^2^)/3
883 reflections	(Δ/σ)_max_ < 0.001
59 parameters	Δρ_max_ = 0.51 e Å^−^^3^
0 restraints	Δρ_min_ = −0.46 e Å^−^^3^
0 constraints	

### (1) Fractional atomic coordinates and isotropic or equivalent isotropic displacement parameters (Å^2^)

**Table:**

	*x*	*y*	*z*	*U*_iso_*/*U*_eq_	
Br1	0.11810 (14)	0.7500	0.05285 (4)	0.0896 (4)	
C2	0.3504 (11)	0.7500	0.1947 (4)	0.0588 (10)	
C3	0.4270 (8)	0.6133 (4)	0.2438 (3)	0.0616 (8)	
C4	0.6007 (9)	0.6158 (3)	0.3467 (3)	0.0615 (7)	
H4A	0.6616	0.5261	0.3814	0.074*	
C5	0.6843 (12)	0.7500	0.3980 (4)	0.0592 (10)	
C6	0.8701 (14)	0.7500	0.5047 (4)	0.0697 (12)	
N7	1.0181 (16)	0.7500	0.5880 (5)	0.0920 (15)	
C8	0.3447 (12)	0.4662 (5)	0.1894 (4)	0.0890 (12)	
H8A	0.4147	0.4671	0.1151	0.133*	
H8B	0.1095	0.4499	0.1878	0.133*	
H8C	0.4573	0.3873	0.2305	0.133*	

### (1) Atomic displacement parameters (Å^2^)

**Table:**

	*U*^11^	*U*^22^	*U*^33^	*U*^12^	*U*^13^	*U*^23^
Br1	0.0749 (5)	0.1338 (7)	0.0588 (5)	0.000	−0.0053 (3)	0.000
C2	0.053 (2)	0.072 (3)	0.052 (2)	0.000	0.0056 (17)	0.000
C3	0.0654 (18)	0.0579 (17)	0.0622 (18)	−0.0049 (13)	0.0085 (14)	−0.0070 (13)
C4	0.0739 (19)	0.0499 (15)	0.0613 (17)	0.0023 (13)	0.0087 (14)	0.0037 (12)
C5	0.067 (3)	0.058 (2)	0.053 (2)	0.000	0.006 (2)	0.000
C6	0.077 (3)	0.075 (3)	0.056 (3)	0.000	−0.001 (2)	0.000
N7	0.104 (4)	0.104 (4)	0.066 (3)	0.000	−0.012 (3)	0.000
C8	0.107 (3)	0.068 (2)	0.092 (3)	−0.015 (2)	0.007 (2)	−0.026 (2)

### (1) Geometric parameters (Å, º)

**Table:**

Br1—C2	1.902 (5)	C5—C6	1.450 (7)
C2—C3	1.384 (4)	C6—N7	1.138 (7)
C3—C4	1.389 (5)	C8—H8A	0.9600
C3—C8	1.498 (5)	C8—H8B	0.9600
C4—C5	1.383 (4)	C8—H8C	0.9600
C4—H4A	0.9300		
			
C3^i^—C2—C3	123.9 (4)	C4—C5—C6	119.8 (2)
C3—C2—Br1	118.1 (2)	N7—C6—C5	179.5 (6)
C2—C3—C4	117.2 (3)	C3—C8—H8A	109.5
C2—C3—C8	123.3 (3)	C3—C8—H8B	109.5
C4—C3—C8	119.5 (3)	H8A—C8—H8B	109.5
C5—C4—C3	120.7 (3)	C3—C8—H8C	109.5
C5—C4—H4A	119.6	H8A—C8—H8C	109.5
C3—C4—H4A	119.6	H8B—C8—H8C	109.5
C4^i^—C5—C4	120.3 (4)		
			
C3^i^—C2—C3—C4	−2.2 (7)	C2—C3—C4—C5	1.6 (5)
Br1—C2—C3—C4	179.1 (3)	C8—C3—C4—C5	179.1 (4)
C3^i^—C2—C3—C8	−179.6 (4)	C3—C4—C5—C4^i^	−1.1 (7)
Br1—C2—C3—C8	1.8 (5)	C3—C4—C5—C6	−179.1 (4)

Symmetry code: (i) *x*, −*y*+3/2, *z*.

### (2) 2-Bromo-1,3-dimethyl-5-nitrobenzene Crystal data

**Table:**

C_8_H_8_BrNO_2_	*F*(000) = 228
*M**_r_* = 230.06	*D*_x_ = 1.716 Mg m^−^^3^
Triclinic, *P*1	Melting point: 478 K
*a* = 4.0502 (5) Å	Cu *K*α radiation, λ = 1.54184 Å
*b* = 9.3817 (6) Å	Cell parameters from 2941 reflections
*c* = 12.1823 (5) Å	θ = 3.7–71.5°
α = 93.498 (4)°	µ = 5.98 mm^−^^1^
β = 99.284 (4)°	*T* = 296 K
γ = 101.722 (5)°	Block, pale yellow
*V* = 445.20 (7) Å^3^	0.80 × 0.60 × 0.10 mm
*Z* = 2	

### (2) 2-Bromo-1,3-dimethyl-5-nitrobenzene Data collection

**Table:**

Rigaku OD SuperNova AtlasS2 diffractometer	1697 independent reflections
Radiation source: micro-focus sealed X-ray tube, SuperNova (Cu) X-ray Source	1503 reflections with *I* > 2σ(*I*)
Mirror monochromator	*R*_int_ = 0.038
Detector resolution: 5.1980 pixels mm^-1^	θ_max_ = 71.7°, θ_min_ = 3.7°
φ and ω scans	*h* = −4→4
Absorption correction: multi-scan (CrysAlis PRO; Rigaku OD, 2015)	*k* = −11→11
*T*_min_ = 0.304, *T*_max_ = 1.000	*l* = −14→14
5640 measured reflections	

### (2) 2-Bromo-1,3-dimethyl-5-nitrobenzene Refinement

**Table:**

Refinement on *F*^2^	Primary atom site location: structure-invariant direct methods
Least-squares matrix: full	Secondary atom site location: difference Fourier map
*R*[*F*^2^ > 2σ(*F*^2^)] = 0.045	Hydrogen site location: inferred from neighbouring sites
*wR*(*F*^2^) = 0.128	H-atom parameters constrained
*S* = 1.07	*w* = 1/[σ^2^(*F*_o_^2^) + (0.0686*P*)^2^ + 0.2116*P*] where *P* = (*F*_o_^2^ + 2*F*_c_^2^)/3
1697 reflections	(Δ/σ)_max_ < 0.001
111 parameters	Δρ_max_ = 0.63 e Å^−^^3^
0 restraints	Δρ_min_ = −0.59 e Å^−^^3^
0 constraints	

### (2) 2-Bromo-1,3-dimethyl-5-nitrobenzene Fractional atomic coordinates and isotropic or equivalent isotropic displacement parameters (Å^2^)

**Table:**

	*x*	*y*	*z*	*U*_iso_*/*U*_eq_	
Br1	0.11600 (12)	0.72052 (5)	0.45994 (3)	0.0787 (2)	
N1	0.2977 (9)	0.7622 (4)	−0.0226 (3)	0.0628 (8)	
O1	0.4131 (11)	0.6691 (4)	−0.0677 (3)	0.0899 (10)	
O2	0.2182 (11)	0.8634 (4)	−0.0707 (3)	0.0947 (11)	
C1	0.1468 (9)	0.8635 (4)	0.2627 (3)	0.0565 (8)	
C2	0.1714 (9)	0.7332 (4)	0.3086 (3)	0.0534 (8)	
C3	0.2324 (9)	0.6117 (4)	0.2500 (3)	0.0548 (8)	
C4	0.2752 (9)	0.6237 (4)	0.1406 (3)	0.0530 (8)	
H4A	0.3211	0.5459	0.0991	0.064*	
C5	0.2495 (9)	0.7520 (4)	0.0932 (3)	0.0533 (8)	
C6	0.1846 (10)	0.8718 (4)	0.1517 (3)	0.0571 (8)	
H6A	0.1667	0.9564	0.1173	0.069*	
C7	0.0874 (12)	0.9962 (5)	0.3270 (4)	0.0779 (12)	
H7A	−0.1068	0.9678	0.3630	0.117*	
H7B	0.0445	1.0676	0.2765	0.117*	
H7C	0.2867	1.0373	0.3824	0.117*	
C8	0.2502 (13)	0.4705 (5)	0.3008 (4)	0.0724 (11)	
H8A	0.4026	0.4907	0.3716	0.109*	
H8B	0.3335	0.4077	0.2517	0.109*	
H8C	0.0257	0.4232	0.3114	0.109*	

### (2) 2-Bromo-1,3-dimethyl-5-nitrobenzene Atomic displacement parameters (Å^2^)

**Table:**

	*U*^11^	*U*^22^	*U*^33^	*U*^12^	*U*^13^	*U*^23^
Br1	0.0850 (4)	0.0998 (4)	0.0536 (3)	0.0207 (3)	0.0197 (2)	0.0041 (2)
N1	0.073 (2)	0.0602 (17)	0.0567 (17)	0.0151 (15)	0.0126 (15)	0.0087 (14)
O1	0.132 (3)	0.093 (2)	0.0637 (18)	0.051 (2)	0.0380 (19)	0.0117 (16)
O2	0.147 (3)	0.084 (2)	0.071 (2)	0.049 (2)	0.031 (2)	0.0291 (17)
C1	0.0481 (17)	0.060 (2)	0.059 (2)	0.0150 (15)	0.0028 (14)	−0.0057 (16)
C2	0.0472 (17)	0.066 (2)	0.0475 (17)	0.0139 (14)	0.0087 (13)	0.0035 (15)
C3	0.0521 (18)	0.0584 (19)	0.0536 (18)	0.0132 (15)	0.0070 (14)	0.0044 (15)
C4	0.0570 (19)	0.0500 (17)	0.0541 (19)	0.0158 (14)	0.0103 (14)	0.0044 (14)
C5	0.0533 (18)	0.0539 (18)	0.0513 (18)	0.0103 (14)	0.0069 (14)	0.0040 (14)
C6	0.061 (2)	0.0497 (18)	0.061 (2)	0.0169 (15)	0.0044 (16)	0.0045 (15)
C7	0.084 (3)	0.072 (3)	0.078 (3)	0.027 (2)	0.010 (2)	−0.016 (2)
C8	0.090 (3)	0.068 (2)	0.064 (2)	0.023 (2)	0.016 (2)	0.0195 (19)

### (2) 2-Bromo-1,3-dimethyl-5-nitrobenzene Geometric parameters (Å, º)

**Table:**

Br1—C2	1.900 (4)	C4—C5	1.382 (5)
N1—O1	1.214 (4)	C4—H4A	0.9300
N1—O2	1.217 (4)	C5—C6	1.388 (5)
N1—C5	1.460 (5)	C6—H6A	0.9300
C1—C6	1.389 (6)	C7—H7A	0.9600
C1—C2	1.390 (5)	C7—H7B	0.9600
C1—C7	1.512 (5)	C7—H7C	0.9600
C2—C3	1.394 (5)	C8—H8A	0.9600
C3—C4	1.379 (5)	C8—H8B	0.9600
C3—C8	1.506 (5)	C8—H8C	0.9600
			
O1—N1—O2	122.2 (4)	C6—C5—N1	118.9 (3)
O1—N1—C5	119.0 (3)	C5—C6—C1	118.9 (3)
O2—N1—C5	118.7 (3)	C5—C6—H6A	120.5
C6—C1—C2	117.7 (3)	C1—C6—H6A	120.5
C6—C1—C7	118.6 (4)	C1—C7—H7A	109.5
C2—C1—C7	123.7 (4)	C1—C7—H7B	109.5
C1—C2—C3	123.8 (3)	H7A—C7—H7B	109.5
C1—C2—Br1	117.9 (3)	C1—C7—H7C	109.5
C3—C2—Br1	118.4 (3)	H7A—C7—H7C	109.5
C4—C3—C2	117.4 (3)	H7B—C7—H7C	109.5
C4—C3—C8	119.8 (3)	C3—C8—H8A	109.5
C2—C3—C8	122.8 (4)	C3—C8—H8B	109.5
C3—C4—C5	119.7 (3)	H8A—C8—H8B	109.5
C3—C4—H4A	120.2	C3—C8—H8C	109.5
C5—C4—H4A	120.2	H8A—C8—H8C	109.5
C4—C5—C6	122.5 (3)	H8B—C8—H8C	109.5
C4—C5—N1	118.6 (3)		
			
C6—C1—C2—C3	0.2 (5)	C3—C4—C5—C6	0.6 (6)
C7—C1—C2—C3	−178.8 (4)	C3—C4—C5—N1	179.7 (3)
C6—C1—C2—Br1	−179.5 (3)	O1—N1—C5—C4	−12.0 (5)
C7—C1—C2—Br1	1.6 (5)	O2—N1—C5—C4	167.5 (4)
C1—C2—C3—C4	1.0 (5)	O1—N1—C5—C6	167.1 (4)
Br1—C2—C3—C4	−179.4 (3)	O2—N1—C5—C6	−13.4 (5)
C1—C2—C3—C8	−178.6 (4)	C4—C5—C6—C1	0.6 (6)
Br1—C2—C3—C8	1.1 (5)	N1—C5—C6—C1	−178.5 (3)
C2—C3—C4—C5	−1.3 (5)	C2—C1—C6—C5	−0.9 (5)
C8—C3—C4—C5	178.2 (3)	C7—C1—C6—C5	178.1 (4)

### (2) 2-Bromo-1,3-dimethyl-5-nitrobenzene Hydrogen-bond geometry (Å, º)

**Table:**

*D*—H···*A*	*D*—H	H···*A*	*D*···*A*	*D*—H···*A*
C4—H4*A*···O1^i^	0.93	2.51	3.377 (5)	156
C6—H6*A*···O2^ii^	0.93	2.55	3.351 (5)	144

Symmetry codes: (i) −*x*+1, −*y*+1, −*z*; (ii) −*x*, −*y*+2, −*z*.

### (3) Crystal data

**Table:**

C_8_H_10_BrN	*D*_x_ = 1.585 Mg m^−^^3^
*M**_r_* = 200.08	Melting point: 346 K
Monoclinic, *P*2_1_/*n*	Cu *K*α radiation, λ = 1.54184 Å
*a* = 10.48314 (15) Å	Cell parameters from 14036 reflections
*b* = 6.10173 (10) Å	θ = 3.4–71.4°
*c* = 26.6195 (5) Å	µ = 6.06 mm^−^^1^
β = 100.0731 (16)°	*T* = 296 K
*V* = 1676.48 (5) Å^3^	Needle, colourless
*Z* = 8	0.30 × 0.12 × 0.10 mm
*F*(000) = 800	

### (3) Data collection

**Table:**

Rigaku OD SuperNova AtlasS2 diffractometer	3276 independent reflections
Radiation source: micro-focus sealed X-ray tube, SuperNova (Cu) X-ray Source	2716 reflections with *I* > 2σ(*I*)
Mirror monochromator	*R*_int_ = 0.093
Detector resolution: 5.1980 pixels mm^-1^	θ_max_ = 72.8°, θ_min_ = 3.4°
φ and ω scans	*h* = −12→12
Absorption correction: multi-scan (CrysAlis PRO; Rigaku OD, 2015)	*k* = −7→7
*T*_min_ = 0.593, *T*_max_ = 1.000	*l* = −32→32
39830 measured reflections	

### (3) Refinement

**Table:**

Refinement on *F*^2^	Primary atom site location: structure-invariant direct methods
Least-squares matrix: full	Secondary atom site location: difference Fourier map
*R*[*F*^2^ > 2σ(*F*^2^)] = 0.054	Hydrogen site location: mixed
*wR*(*F*^2^) = 0.165	H atoms treated by a mixture of independent and constrained refinement
*S* = 1.11	*w* = 1/[σ^2^(*F*_o_^2^) + (0.0779*P*)^2^ + 1.0194*P*] where *P* = (*F*_o_^2^ + 2*F*_c_^2^)/3
3276 reflections	(Δ/σ)_max_ < 0.001
197 parameters	Δρ_max_ = 0.44 e Å^−^^3^
6 restraints	Δρ_min_ = −1.11 e Å^−^^3^
0 constraints	

### (3) Fractional atomic coordinates and isotropic or equivalent isotropic displacement parameters (Å^2^)

**Table:**

	*x*	*y*	*z*	*U*_iso_*/*U*_eq_	
Br1	0.24694 (5)	0.23484 (10)	0.15702 (3)	0.1038 (3)	
N1	−0.1935 (4)	0.7889 (7)	0.20733 (16)	0.0868 (12)	
H1A	−0.201 (5)	0.787 (7)	0.2398 (9)	0.104*	
H1B	−0.202 (5)	0.918 (5)	0.1918 (15)	0.104*	
C1	0.0703 (3)	0.5931 (7)	0.14294 (14)	0.0673 (9)	
C2	0.1125 (3)	0.4160 (6)	0.17418 (14)	0.0618 (8)	
C3	0.0613 (3)	0.3673 (6)	0.21759 (14)	0.0616 (8)	
C4	−0.0384 (3)	0.4981 (6)	0.22843 (12)	0.0601 (8)	
H4A	−0.0745	0.4684	0.2572	0.072*	
C5	−0.0852 (3)	0.6715 (7)	0.19735 (13)	0.0608 (8)	
C6	−0.0292 (4)	0.7188 (6)	0.15532 (15)	0.0673 (9)	
H6A	−0.0593	0.8382	0.1349	0.081*	
C7	0.1289 (6)	0.6536 (10)	0.0969 (2)	0.1049 (17)	
H7A	0.2212	0.6663	0.1067	0.157*	
H7B	0.0938	0.7910	0.0834	0.157*	
H7C	0.1091	0.5419	0.0713	0.157*	
C8	0.1109 (5)	0.1815 (8)	0.2535 (2)	0.0951 (14)	
H8A	0.1029	0.0456	0.2351	0.143*	
H8B	0.0611	0.1745	0.2805	0.143*	
H8C	0.2003	0.2068	0.2678	0.143*	
Br11	0.72828 (6)	−0.22871 (10)	−0.00212 (2)	0.0996 (3)	
N11	0.6186 (4)	0.3496 (7)	0.17122 (14)	0.0816 (10)	
H11A	0.658 (4)	0.480 (5)	0.1713 (19)	0.098*	
H11B	0.536 (2)	0.356 (7)	0.1745 (18)	0.098*	
C11	0.5864 (3)	−0.0934 (7)	0.07484 (15)	0.0681 (9)	
C12	0.6933 (3)	−0.0488 (7)	0.05224 (13)	0.0652 (9)	
C13	0.7753 (3)	0.1285 (7)	0.06764 (13)	0.0652 (9)	
C14	0.7478 (4)	0.2596 (6)	0.10694 (14)	0.0648 (9)	
H14A	0.8018	0.3773	0.1180	0.078*	
C15	0.6412 (4)	0.2187 (7)	0.13007 (14)	0.0649 (9)	
C16	0.5626 (3)	0.0413 (7)	0.11379 (14)	0.0688 (10)	
H16A	0.4918	0.0122	0.1295	0.083*	
C17	0.4956 (5)	−0.2818 (8)	0.0571 (2)	0.0917 (14)	
H17A	0.4340	−0.2971	0.0796	0.138*	
H17B	0.5447	−0.4147	0.0572	0.138*	
H17C	0.4507	−0.2530	0.0231	0.138*	
C18	0.8907 (5)	0.1869 (9)	0.04352 (18)	0.0884 (13)	
H18A	0.8643	0.1973	0.0072	0.133*	
H18B	0.9558	0.0755	0.0513	0.133*	
H18C	0.9254	0.3251	0.0567	0.133*	

### (3) Atomic displacement parameters (Å^2^)

**Table:**

	*U*^11^	*U*^22^	*U*^33^	*U*^12^	*U*^13^	*U*^23^
Br1	0.0617 (3)	0.1074 (5)	0.1481 (6)	0.0103 (2)	0.0348 (3)	−0.0438 (3)
N1	0.066 (2)	0.117 (3)	0.082 (2)	0.034 (2)	0.0268 (18)	0.008 (2)
C1	0.0627 (19)	0.081 (2)	0.066 (2)	−0.0104 (18)	0.0315 (16)	−0.0087 (18)
C2	0.0408 (14)	0.068 (2)	0.080 (2)	0.0016 (14)	0.0206 (14)	−0.0159 (18)
C3	0.0466 (16)	0.065 (2)	0.072 (2)	−0.0036 (14)	0.0078 (14)	0.0020 (17)
C4	0.0478 (16)	0.079 (2)	0.0569 (17)	−0.0032 (15)	0.0185 (13)	0.0057 (17)
C5	0.0479 (16)	0.079 (2)	0.0584 (18)	0.0061 (16)	0.0183 (14)	−0.0009 (17)
C6	0.071 (2)	0.077 (2)	0.0575 (19)	0.0061 (18)	0.0207 (16)	0.0090 (17)
C7	0.122 (4)	0.118 (4)	0.093 (3)	−0.022 (3)	0.070 (3)	−0.009 (3)
C8	0.099 (3)	0.079 (3)	0.104 (4)	0.014 (3)	0.008 (3)	0.018 (3)
Br11	0.1085 (5)	0.1057 (5)	0.0899 (4)	0.0198 (3)	0.0326 (3)	−0.0249 (3)
N11	0.075 (2)	0.105 (3)	0.073 (2)	0.000 (2)	0.0369 (17)	−0.018 (2)
C11	0.0536 (17)	0.077 (2)	0.072 (2)	0.0145 (17)	0.0074 (15)	0.0043 (19)
C12	0.0644 (19)	0.078 (2)	0.0551 (17)	0.0193 (18)	0.0154 (15)	0.0002 (17)
C13	0.0575 (18)	0.086 (3)	0.0557 (18)	0.0144 (18)	0.0199 (15)	0.0073 (18)
C14	0.059 (2)	0.081 (3)	0.059 (2)	0.0026 (16)	0.0219 (16)	0.0020 (17)
C15	0.0562 (19)	0.085 (3)	0.0566 (18)	0.0134 (17)	0.0197 (15)	0.0017 (17)
C16	0.0485 (17)	0.090 (3)	0.072 (2)	0.0096 (17)	0.0224 (15)	0.006 (2)
C17	0.072 (3)	0.089 (3)	0.114 (4)	−0.003 (2)	0.014 (3)	−0.007 (3)
C18	0.084 (3)	0.115 (4)	0.078 (3)	0.003 (3)	0.046 (2)	0.001 (3)

### (3) Geometric parameters (Å, º)

**Table:**

Br1—C2	1.908 (3)	Br11—C12	1.902 (3)
N1—C5	1.407 (5)	N11—C15	1.409 (5)
N1—H1A	0.881 (18)	N11—H11A	0.897 (18)
N1—H1B	0.886 (18)	N11—H11B	0.884 (18)
C1—C6	1.380 (5)	C11—C16	1.380 (5)
C1—C2	1.388 (6)	C11—C12	1.389 (5)
C1—C7	1.510 (5)	C11—C17	1.514 (6)
C2—C3	1.389 (5)	C12—C13	1.398 (6)
C3—C4	1.385 (5)	C13—C14	1.387 (5)
C3—C8	1.515 (6)	C13—C18	1.508 (5)
C4—C5	1.379 (5)	C14—C15	1.390 (5)
C4—H4A	0.9300	C14—H14A	0.9300
C5—C6	1.382 (5)	C15—C16	1.383 (6)
C6—H6A	0.9300	C16—H16A	0.9300
C7—H7A	0.9600	C17—H17A	0.9600
C7—H7B	0.9600	C17—H17B	0.9600
C7—H7C	0.9600	C17—H17C	0.9600
C8—H8A	0.9600	C18—H18A	0.9600
C8—H8B	0.9600	C18—H18B	0.9600
C8—H8C	0.9600	C18—H18C	0.9600
			
C5—N1—H1A	113 (3)	C15—N11—H11A	111 (3)
C5—N1—H1B	113 (3)	C15—N11—H11B	114 (3)
H1A—N1—H1B	117 (3)	H11A—N11—H11B	115 (3)
C6—C1—C2	117.7 (3)	C16—C11—C12	118.3 (4)
C6—C1—C7	119.3 (4)	C16—C11—C17	120.0 (4)
C2—C1—C7	123.0 (4)	C12—C11—C17	121.6 (4)
C1—C2—C3	122.4 (3)	C11—C12—C13	121.8 (3)
C1—C2—Br1	118.6 (3)	C11—C12—Br11	119.6 (3)
C3—C2—Br1	119.0 (3)	C13—C12—Br11	118.6 (3)
C4—C3—C2	117.8 (3)	C14—C13—C12	118.0 (3)
C4—C3—C8	119.2 (4)	C14—C13—C18	118.2 (4)
C2—C3—C8	123.0 (4)	C12—C13—C18	123.8 (4)
C5—C4—C3	121.3 (3)	C13—C14—C15	121.3 (4)
C5—C4—H4A	119.4	C13—C14—H14A	119.3
C3—C4—H4A	119.4	C15—C14—H14A	119.3
C4—C5—C6	119.3 (3)	C16—C15—C14	118.9 (3)
C4—C5—N1	119.5 (3)	C16—C15—N11	121.0 (3)
C6—C5—N1	121.2 (4)	C14—C15—N11	120.0 (4)
C1—C6—C5	121.5 (4)	C11—C16—C15	121.7 (3)
C1—C6—H6A	119.2	C11—C16—H16A	119.2
C5—C6—H6A	119.2	C15—C16—H16A	119.2
C1—C7—H7A	109.5	C11—C17—H17A	109.5
C1—C7—H7B	109.5	C11—C17—H17B	109.5
H7A—C7—H7B	109.5	H17A—C17—H17B	109.5
C1—C7—H7C	109.5	C11—C17—H17C	109.5
H7A—C7—H7C	109.5	H17A—C17—H17C	109.5
H7B—C7—H7C	109.5	H17B—C17—H17C	109.5
C3—C8—H8A	109.5	C13—C18—H18A	109.5
C3—C8—H8B	109.5	C13—C18—H18B	109.5
H8A—C8—H8B	109.5	H18A—C18—H18B	109.5
C3—C8—H8C	109.5	C13—C18—H18C	109.5
H8A—C8—H8C	109.5	H18A—C18—H18C	109.5
H8B—C8—H8C	109.5	H18B—C18—H18C	109.5
			
C6—C1—C2—C3	−2.1 (6)	C16—C11—C12—C13	−0.6 (5)
C7—C1—C2—C3	177.4 (4)	C17—C11—C12—C13	178.3 (4)
C6—C1—C2—Br1	178.4 (3)	C16—C11—C12—Br11	−179.3 (3)
C7—C1—C2—Br1	−2.1 (5)	C17—C11—C12—Br11	−0.4 (5)
C1—C2—C3—C4	2.1 (5)	C11—C12—C13—C14	0.7 (5)
Br1—C2—C3—C4	−178.4 (3)	Br11—C12—C13—C14	179.3 (3)
C1—C2—C3—C8	−176.8 (4)	C11—C12—C13—C18	−178.5 (4)
Br1—C2—C3—C8	2.6 (5)	Br11—C12—C13—C18	0.2 (5)
C2—C3—C4—C5	−0.2 (5)	C12—C13—C14—C15	−0.8 (6)
C8—C3—C4—C5	178.8 (4)	C18—C13—C14—C15	178.4 (4)
C3—C4—C5—C6	−1.7 (6)	C13—C14—C15—C16	0.9 (6)
C3—C4—C5—N1	174.7 (4)	C13—C14—C15—N11	177.6 (4)
C2—C1—C6—C5	0.1 (6)	C12—C11—C16—C15	0.8 (6)
C7—C1—C6—C5	−179.4 (4)	C17—C11—C16—C15	−178.2 (4)
C4—C5—C6—C1	1.7 (6)	C14—C15—C16—C11	−0.9 (6)
N1—C5—C6—C1	−174.6 (4)	N11—C15—C16—C11	−177.5 (4)

### (3) Hydrogen-bond geometry (Å, º)

**Table:**

*D*—H···*A*	*D*—H	H···*A*	*D*···*A*	*D*—H···*A*
N1—H1*A*···N11^i^	0.88 (2)	2.41 (3)	3.212 (6)	152 (5)
N11—H11*A*···N1^ii^	0.90 (2)	2.52 (3)	3.365 (6)	157 (4)

Symmetry codes: (i) −*x*+1/2, *y*+1/2, −*z*+1/2; (ii) *x*+1, *y*, *z*.

### (4) 4-Bromo-3,5-dimethylphenol Crystal data

**Table:**

C_8_H_9_BrO	*D*_x_ = 1.692 Mg m^−^^3^
*M**_r_* = 201.06	Melting point: 386 K
Orthorhombic, *P**b**c**a*	Cu *K*α radiation, λ = 1.54184 Å
*a* = 14.65213 (17) Å	Cell parameters from 10448 reflections
*b* = 17.9520 (2) Å	θ = 3.7–71.4°
*c* = 24.0079 (3) Å	µ = 6.50 mm^−^^1^
*V* = 6314.94 (12) Å^3^	*T* = 100 K
*Z* = 32	Block, yellow
*F*(000) = 3200	0.23 × 0.20 × 0.18 mm

### (4) 4-Bromo-3,5-dimethylphenol Data collection

**Table:**

Rigaku OD SuperNova AtlasS2 diffractometer	6110 independent reflections
Radiation source: micro-focus sealed X-ray tube, SuperNova (Cu) X-ray Source	5342 reflections with *I* > 2σ(*I*)
Mirror monochromator	*R*_int_ = 0.033
Detector resolution: 5.1980 pixels mm^-1^	θ_max_ = 71.6°, θ_min_ = 3.7°
φ and ω scans	*h* = −17→13
Absorption correction: multi-scan (CrysAlis PRO; Rigaku OD, 2015)	*k* = −21→22
*T*_min_ = 0.601, *T*_max_ = 1.000	*l* = −29→28
22136 measured reflections	

### (4) 4-Bromo-3,5-dimethylphenol Refinement

**Table:**

Refinement on *F*^2^	Primary atom site location: structure-invariant direct methods
Least-squares matrix: full	Secondary atom site location: difference Fourier map
*R*[*F*^2^ > 2σ(*F*^2^)] = 0.026	Hydrogen site location: mixed
*wR*(*F*^2^) = 0.065	H atoms treated by a mixture of independent and constrained refinement
*S* = 1.02	*w* = 1/[σ^2^(*F*_o_^2^) + (0.031*P*)^2^ + 3.051*P*] where *P* = (*F*_o_^2^ + 2*F*_c_^2^)/3
6110 reflections	(Δ/σ)_max_ = 0.002
381 parameters	Δρ_max_ = 0.56 e Å^−^^3^
0 restraints	Δρ_min_ = −0.43 e Å^−^^3^
0 constraints	

### (4) 4-Bromo-3,5-dimethylphenol Fractional atomic coordinates and isotropic or equivalent isotropic displacement parameters (Å^2^)

**Table:**

	*x*	*y*	*z*	*U*_iso_*/*U*_eq_	
Br1	0.22468 (2)	0.59654 (2)	0.78001 (2)	0.02219 (7)	
O1	0.46483 (12)	0.75860 (10)	0.61587 (8)	0.0196 (4)	
H1	0.436 (2)	0.7751 (18)	0.5913 (14)	0.029*	
C1	0.25870 (16)	0.69235 (13)	0.68779 (10)	0.0161 (5)	
C2	0.30052 (17)	0.65022 (13)	0.72925 (10)	0.0162 (5)	
C3	0.39536 (17)	0.64366 (13)	0.73399 (10)	0.0159 (5)	
C4	0.44884 (16)	0.68140 (13)	0.69532 (10)	0.0151 (5)	
H4A	0.5135	0.6785	0.6976	0.018*	
C5	0.40869 (16)	0.72306 (13)	0.65365 (10)	0.0145 (5)	
C6	0.31470 (16)	0.72865 (13)	0.64976 (10)	0.0164 (5)	
H6A	0.2882	0.7576	0.6208	0.020*	
C7	0.15740 (17)	0.69908 (16)	0.68248 (11)	0.0234 (6)	
H7A	0.1318	0.7172	0.7177	0.035*	
H7B	0.1313	0.6502	0.6737	0.035*	
H7C	0.1427	0.7342	0.6526	0.035*	
C8	0.44007 (18)	0.59541 (14)	0.77743 (11)	0.0219 (5)	
H8A	0.5061	0.5944	0.7711	0.033*	
H8B	0.4157	0.5447	0.7749	0.033*	
H8C	0.4274	0.6157	0.8145	0.033*	
Br11	0.90409 (2)	0.88537 (2)	0.75012 (2)	0.02282 (7)	
O11	0.64155 (12)	0.73224 (11)	0.59470 (8)	0.0230 (4)	
H11	0.590 (2)	0.7371 (19)	0.6026 (14)	0.034*	
C11	0.72728 (16)	0.84371 (13)	0.71234 (10)	0.0159 (5)	
C12	0.81977 (16)	0.83541 (13)	0.70215 (10)	0.0151 (5)	
C13	0.85456 (16)	0.79230 (13)	0.65883 (10)	0.0152 (5)	
C14	0.79235 (16)	0.75794 (14)	0.62345 (10)	0.0164 (5)	
H14A	0.8137	0.7281	0.5935	0.020*	
C15	0.69938 (16)	0.76692 (13)	0.63161 (10)	0.0146 (5)	
C16	0.66644 (16)	0.80871 (13)	0.67584 (10)	0.0145 (5)	
H16A	0.6025	0.8136	0.6814	0.017*	
C17	0.69077 (19)	0.88894 (15)	0.76024 (11)	0.0230 (6)	
H17A	0.7141	0.8687	0.7954	0.034*	
H17B	0.6239	0.8867	0.7603	0.034*	
H17C	0.7105	0.9408	0.7563	0.034*	
C18	0.95504 (16)	0.78010 (15)	0.65044 (11)	0.0207 (5)	
H18A	0.9646	0.7475	0.6182	0.031*	
H18B	0.9810	0.7567	0.6838	0.031*	
H18C	0.9851	0.8281	0.6439	0.031*	
Br21	0.42678 (2)	0.47389 (2)	0.39094 (2)	0.02459 (7)	
O21	0.69191 (12)	0.69370 (10)	0.49124 (7)	0.0175 (4)	
H21	0.677 (2)	0.7063 (18)	0.5200 (13)	0.026*	
C21	0.47716 (16)	0.59328 (14)	0.46321 (10)	0.0167 (5)	
C22	0.51005 (17)	0.54353 (13)	0.42327 (10)	0.0175 (5)	
C23	0.60053 (17)	0.54233 (13)	0.40577 (10)	0.0167 (5)	
C24	0.66009 (17)	0.59418 (13)	0.42934 (10)	0.0163 (5)	
H24A	0.7221	0.5953	0.4179	0.020*	
C25	0.62964 (16)	0.64384 (13)	0.46920 (10)	0.0144 (5)	
C26	0.53944 (16)	0.64363 (13)	0.48600 (10)	0.0158 (5)	
H26A	0.5195	0.6782	0.5134	0.019*	
C27	0.37892 (17)	0.59441 (16)	0.48179 (11)	0.0233 (6)	
H27A	0.3712	0.6316	0.5113	0.035*	
H27B	0.3620	0.5452	0.4960	0.035*	
H27C	0.3397	0.6072	0.4501	0.035*	
C28	0.63566 (19)	0.48676 (14)	0.36405 (11)	0.0221 (5)	
H28A	0.6999	0.4974	0.3558	0.033*	
H28B	0.5998	0.4903	0.3297	0.033*	
H28C	0.6302	0.4364	0.3794	0.033*	
Br31	1.07251 (2)	0.46757 (2)	0.62030 (2)	0.02168 (7)	
O31	0.87558 (12)	0.69132 (10)	0.47660 (7)	0.0176 (4)	
H31	0.823 (2)	0.6897 (17)	0.4800 (13)	0.026*	
C31	0.91446 (17)	0.53218 (13)	0.57080 (10)	0.0154 (5)	
C32	1.00912 (17)	0.53671 (13)	0.57385 (10)	0.0155 (5)	
C33	1.06015 (16)	0.59008 (14)	0.54511 (10)	0.0158 (5)	
C34	1.01266 (16)	0.64133 (13)	0.51263 (10)	0.0152 (5)	
H34A	1.0451	0.6784	0.4925	0.018*	
C35	0.91818 (16)	0.63854 (13)	0.50948 (10)	0.0139 (5)	
C36	0.86927 (16)	0.58457 (13)	0.53791 (10)	0.0147 (5)	
H36A	0.8046	0.5832	0.5350	0.018*	
C37	0.86046 (18)	0.47289 (13)	0.60069 (11)	0.0200 (5)	
H37A	0.8724	0.4760	0.6408	0.030*	
H37B	0.8787	0.4237	0.5870	0.030*	
H37C	0.7952	0.4804	0.5937	0.030*	
C38	1.16258 (16)	0.59329 (15)	0.54722 (11)	0.0217 (5)	
H38A	1.1841	0.6362	0.5256	0.033*	
H38B	1.1879	0.5474	0.5314	0.033*	
H38C	1.1825	0.5984	0.5860	0.033*	

### (4) 4-Bromo-3,5-dimethylphenol Atomic displacement parameters (Å^2^)

**Table:**

	*U*^11^	*U*^22^	*U*^33^	*U*^12^	*U*^13^	*U*^23^
Br1	0.02410 (15)	0.02111 (14)	0.02136 (14)	−0.00353 (10)	0.00837 (10)	0.00205 (10)
O1	0.0138 (9)	0.0259 (10)	0.0191 (9)	−0.0019 (7)	−0.0014 (7)	0.0086 (7)
C1	0.0150 (12)	0.0148 (11)	0.0187 (12)	0.0004 (9)	0.0032 (9)	−0.0035 (9)
C2	0.0195 (12)	0.0137 (11)	0.0155 (11)	−0.0036 (9)	0.0044 (9)	−0.0027 (9)
C3	0.0212 (13)	0.0125 (11)	0.0139 (11)	−0.0008 (9)	−0.0033 (9)	−0.0019 (9)
C4	0.0115 (11)	0.0152 (11)	0.0187 (12)	−0.0007 (9)	−0.0012 (9)	−0.0021 (9)
C5	0.0134 (11)	0.0144 (11)	0.0156 (11)	−0.0028 (9)	0.0013 (9)	0.0000 (9)
C6	0.0189 (12)	0.0161 (12)	0.0141 (11)	0.0021 (9)	−0.0019 (9)	0.0009 (9)
C7	0.0162 (13)	0.0303 (15)	0.0238 (13)	0.0005 (11)	0.0036 (10)	−0.0007 (11)
C8	0.0244 (14)	0.0216 (13)	0.0198 (13)	−0.0047 (10)	−0.0062 (10)	0.0041 (11)
Br11	0.02236 (14)	0.02426 (14)	0.02183 (14)	−0.00606 (10)	−0.00779 (10)	−0.00253 (11)
O11	0.0123 (9)	0.0369 (11)	0.0197 (9)	−0.0031 (8)	−0.0010 (7)	−0.0103 (8)
C11	0.0207 (13)	0.0113 (11)	0.0158 (11)	0.0024 (9)	0.0002 (9)	0.0014 (9)
C12	0.0164 (12)	0.0145 (11)	0.0145 (11)	−0.0021 (9)	−0.0044 (9)	0.0016 (9)
C13	0.0131 (12)	0.0169 (12)	0.0156 (11)	−0.0007 (9)	−0.0017 (9)	0.0033 (9)
C14	0.0166 (12)	0.0189 (12)	0.0136 (11)	0.0009 (9)	0.0013 (9)	−0.0036 (9)
C15	0.0142 (12)	0.0157 (11)	0.0139 (11)	−0.0030 (9)	−0.0023 (9)	−0.0004 (9)
C16	0.0121 (11)	0.0150 (11)	0.0163 (11)	0.0013 (9)	0.0000 (9)	0.0019 (9)
C17	0.0261 (14)	0.0223 (13)	0.0205 (13)	0.0005 (11)	0.0016 (11)	−0.0068 (10)
C18	0.0141 (12)	0.0274 (13)	0.0206 (13)	0.0002 (10)	0.0011 (10)	0.0021 (10)
Br21	0.02642 (15)	0.02282 (14)	0.02452 (15)	−0.00989 (11)	−0.00721 (11)	−0.00050 (11)
O21	0.0134 (8)	0.0209 (9)	0.0182 (9)	−0.0034 (7)	0.0007 (7)	−0.0071 (7)
C21	0.0153 (12)	0.0186 (12)	0.0163 (12)	−0.0024 (9)	−0.0006 (9)	0.0057 (10)
C22	0.0196 (13)	0.0158 (11)	0.0170 (12)	−0.0046 (9)	−0.0050 (10)	0.0043 (9)
C23	0.0235 (13)	0.0142 (11)	0.0122 (11)	0.0005 (10)	−0.0019 (10)	0.0014 (9)
C24	0.0164 (12)	0.0174 (12)	0.0151 (11)	−0.0016 (9)	0.0005 (9)	0.0020 (9)
C25	0.0157 (12)	0.0133 (11)	0.0142 (11)	−0.0024 (9)	−0.0050 (9)	0.0015 (9)
C26	0.0145 (12)	0.0174 (11)	0.0156 (11)	0.0011 (9)	−0.0008 (9)	−0.0009 (9)
C27	0.0140 (13)	0.0306 (14)	0.0252 (14)	−0.0046 (10)	0.0000 (10)	0.0023 (11)
C28	0.0299 (15)	0.0174 (12)	0.0191 (13)	−0.0002 (11)	0.0006 (11)	−0.0035 (10)
Br31	0.02599 (15)	0.02241 (14)	0.01663 (13)	0.00797 (10)	−0.00221 (10)	0.00384 (10)
O31	0.0120 (8)	0.0197 (9)	0.0211 (9)	0.0029 (7)	0.0013 (7)	0.0071 (7)
C31	0.0226 (13)	0.0134 (11)	0.0101 (11)	−0.0013 (9)	0.0026 (9)	−0.0020 (9)
C32	0.0206 (12)	0.0155 (12)	0.0104 (11)	0.0045 (9)	−0.0003 (9)	−0.0011 (9)
C33	0.0163 (12)	0.0178 (12)	0.0133 (11)	0.0002 (9)	−0.0008 (9)	−0.0031 (9)
C34	0.0148 (12)	0.0147 (11)	0.0160 (11)	0.0003 (9)	0.0028 (9)	0.0002 (9)
C35	0.0156 (12)	0.0142 (11)	0.0119 (11)	0.0009 (9)	−0.0009 (9)	−0.0001 (9)
C36	0.0139 (12)	0.0168 (12)	0.0133 (11)	−0.0010 (9)	0.0005 (9)	−0.0006 (9)
C37	0.0249 (14)	0.0173 (12)	0.0178 (12)	−0.0030 (10)	0.0026 (10)	0.0033 (10)
C38	0.0135 (12)	0.0265 (14)	0.0252 (14)	0.0020 (10)	−0.0009 (10)	−0.0016 (11)

### (4) 4-Bromo-3,5-dimethylphenol Geometric parameters (Å, º)

**Table:**

Br1—C2	1.910 (2)	Br21—C22	1.912 (2)
O1—C5	1.381 (3)	O21—C25	1.383 (3)
O1—H1	0.78 (3)	O21—H21	0.76 (3)
C1—C6	1.390 (3)	C21—C22	1.396 (4)
C1—C2	1.392 (3)	C21—C26	1.396 (3)
C1—C7	1.495 (3)	C21—C27	1.507 (3)
C2—C3	1.399 (3)	C22—C23	1.391 (4)
C3—C4	1.391 (3)	C23—C24	1.396 (3)
C3—C8	1.506 (3)	C23—C28	1.504 (3)
C4—C5	1.381 (3)	C24—C25	1.382 (3)
C4—H4A	0.9500	C24—H24A	0.9500
C5—C6	1.384 (3)	C25—C26	1.382 (3)
C6—H6A	0.9500	C26—H26A	0.9500
C7—H7A	0.9800	C27—H27A	0.9800
C7—H7B	0.9800	C27—H27B	0.9800
C7—H7C	0.9800	C27—H27C	0.9800
C8—H8A	0.9800	C28—H28A	0.9800
C8—H8B	0.9800	C28—H28B	0.9800
C8—H8C	0.9800	C28—H28C	0.9800
Br11—C12	1.912 (2)	Br31—C32	1.910 (2)
O11—C15	1.375 (3)	O31—C35	1.382 (3)
O11—H11	0.78 (3)	O31—H31	0.77 (3)
C11—C12	1.385 (3)	C31—C32	1.391 (3)
C11—C16	1.399 (3)	C31—C36	1.395 (3)
C11—C17	1.506 (3)	C31—C37	1.508 (3)
C12—C13	1.393 (3)	C32—C33	1.398 (3)
C13—C14	1.391 (3)	C33—C34	1.392 (3)
C13—C18	1.502 (3)	C33—C38	1.503 (3)
C14—C15	1.386 (3)	C34—C35	1.387 (3)
C14—H14A	0.9500	C34—H34A	0.9500
C15—C16	1.387 (3)	C35—C36	1.385 (3)
C16—H16A	0.9500	C36—H36A	0.9500
C17—H17A	0.9800	C37—H37A	0.9800
C17—H17B	0.9800	C37—H37B	0.9800
C17—H17C	0.9800	C37—H37C	0.9800
C18—H18A	0.9800	C38—H38A	0.9800
C18—H18B	0.9800	C38—H38B	0.9800
C18—H18C	0.9800	C38—H38C	0.9800
			
C5—O1—H1	110 (2)	C25—O21—H21	111 (2)
C6—C1—C2	117.7 (2)	C22—C21—C26	117.2 (2)
C6—C1—C7	119.5 (2)	C22—C21—C27	122.8 (2)
C2—C1—C7	122.8 (2)	C26—C21—C27	120.0 (2)
C1—C2—C3	122.7 (2)	C23—C22—C21	123.1 (2)
C1—C2—Br1	118.31 (18)	C23—C22—Br21	118.39 (19)
C3—C2—Br1	118.90 (18)	C21—C22—Br21	118.48 (18)
C4—C3—C2	117.7 (2)	C22—C23—C24	117.6 (2)
C4—C3—C8	119.8 (2)	C22—C23—C28	122.5 (2)
C2—C3—C8	122.5 (2)	C24—C23—C28	119.9 (2)
C5—C4—C3	120.5 (2)	C25—C24—C23	120.6 (2)
C5—C4—H4A	119.8	C25—C24—H24A	119.7
C3—C4—H4A	119.8	C23—C24—H24A	119.7
C4—C5—O1	118.2 (2)	C26—C25—C24	120.6 (2)
C4—C5—C6	120.8 (2)	C26—C25—O21	121.4 (2)
O1—C5—C6	121.0 (2)	C24—C25—O21	118.0 (2)
C5—C6—C1	120.6 (2)	C25—C26—C21	120.8 (2)
C5—C6—H6A	119.7	C25—C26—H26A	119.6
C1—C6—H6A	119.7	C21—C26—H26A	119.6
C1—C7—H7A	109.5	C21—C27—H27A	109.5
C1—C7—H7B	109.5	C21—C27—H27B	109.5
H7A—C7—H7B	109.5	H27A—C27—H27B	109.5
C1—C7—H7C	109.5	C21—C27—H27C	109.5
H7A—C7—H7C	109.5	H27A—C27—H27C	109.5
H7B—C7—H7C	109.5	H27B—C27—H27C	109.5
C3—C8—H8A	109.5	C23—C28—H28A	109.5
C3—C8—H8B	109.5	C23—C28—H28B	109.5
H8A—C8—H8B	109.5	H28A—C28—H28B	109.5
C3—C8—H8C	109.5	C23—C28—H28C	109.5
H8A—C8—H8C	109.5	H28A—C28—H28C	109.5
H8B—C8—H8C	109.5	H28B—C28—H28C	109.5
C15—O11—H11	113 (3)	C35—O31—H31	111 (2)
C12—C11—C16	117.7 (2)	C32—C31—C36	117.6 (2)
C12—C11—C17	122.7 (2)	C32—C31—C37	122.6 (2)
C16—C11—C17	119.6 (2)	C36—C31—C37	119.7 (2)
C11—C12—C13	123.3 (2)	C31—C32—C33	123.2 (2)
C11—C12—Br11	118.38 (18)	C31—C32—Br31	118.53 (18)
C13—C12—Br11	118.27 (18)	C33—C32—Br31	118.27 (18)
C14—C13—C12	117.6 (2)	C34—C33—C32	117.5 (2)
C14—C13—C18	119.7 (2)	C34—C33—C38	119.5 (2)
C12—C13—C18	122.7 (2)	C32—C33—C38	123.0 (2)
C15—C14—C13	120.4 (2)	C35—C34—C33	120.4 (2)
C15—C14—H14A	119.8	C35—C34—H34A	119.8
C13—C14—H14A	119.8	C33—C34—H34A	119.8
O11—C15—C14	117.5 (2)	O31—C35—C36	121.8 (2)
O11—C15—C16	121.6 (2)	O31—C35—C34	117.2 (2)
C14—C15—C16	120.9 (2)	C36—C35—C34	121.0 (2)
C15—C16—C11	120.0 (2)	C35—C36—C31	120.3 (2)
C15—C16—H16A	120.0	C35—C36—H36A	119.8
C11—C16—H16A	120.0	C31—C36—H36A	119.8
C11—C17—H17A	109.5	C31—C37—H37A	109.5
C11—C17—H17B	109.5	C31—C37—H37B	109.5
H17A—C17—H17B	109.5	H37A—C37—H37B	109.5
C11—C17—H17C	109.5	C31—C37—H37C	109.5
H17A—C17—H17C	109.5	H37A—C37—H37C	109.5
H17B—C17—H17C	109.5	H37B—C37—H37C	109.5
C13—C18—H18A	109.5	C33—C38—H38A	109.5
C13—C18—H18B	109.5	C33—C38—H38B	109.5
H18A—C18—H18B	109.5	H38A—C38—H38B	109.5
C13—C18—H18C	109.5	C33—C38—H38C	109.5
H18A—C18—H18C	109.5	H38A—C38—H38C	109.5
H18B—C18—H18C	109.5	H38B—C38—H38C	109.5
			
C6—C1—C2—C3	0.3 (4)	C26—C21—C22—C23	0.0 (4)
C7—C1—C2—C3	179.6 (2)	C27—C21—C22—C23	179.8 (2)
C6—C1—C2—Br1	−177.41 (17)	C26—C21—C22—Br21	−179.83 (17)
C7—C1—C2—Br1	1.9 (3)	C27—C21—C22—Br21	−0.1 (3)
C1—C2—C3—C4	0.2 (4)	C21—C22—C23—C24	−0.6 (4)
Br1—C2—C3—C4	177.91 (17)	Br21—C22—C23—C24	179.23 (17)
C1—C2—C3—C8	−177.3 (2)	C21—C22—C23—C28	178.0 (2)
Br1—C2—C3—C8	0.4 (3)	Br21—C22—C23—C28	−2.2 (3)
C2—C3—C4—C5	−0.6 (3)	C22—C23—C24—C25	1.0 (3)
C8—C3—C4—C5	177.0 (2)	C28—C23—C24—C25	−177.7 (2)
C3—C4—C5—O1	−179.1 (2)	C23—C24—C25—C26	−0.7 (4)
C3—C4—C5—C6	0.6 (4)	C23—C24—C25—O21	179.7 (2)
C4—C5—C6—C1	0.0 (4)	C24—C25—C26—C21	0.1 (4)
O1—C5—C6—C1	179.6 (2)	O21—C25—C26—C21	179.6 (2)
C2—C1—C6—C5	−0.4 (3)	C22—C21—C26—C25	0.3 (3)
C7—C1—C6—C5	−179.7 (2)	C27—C21—C26—C25	−179.5 (2)
C16—C11—C12—C13	−2.2 (4)	C36—C31—C32—C33	−1.0 (4)
C17—C11—C12—C13	178.4 (2)	C37—C31—C32—C33	178.1 (2)
C16—C11—C12—Br11	178.05 (17)	C36—C31—C32—Br31	178.17 (17)
C17—C11—C12—Br11	−1.4 (3)	C37—C31—C32—Br31	−2.7 (3)
C11—C12—C13—C14	1.9 (4)	C31—C32—C33—C34	0.8 (4)
Br11—C12—C13—C14	−178.34 (18)	Br31—C32—C33—C34	−178.28 (17)
C11—C12—C13—C18	−176.2 (2)	C31—C32—C33—C38	−178.1 (2)
Br11—C12—C13—C18	3.6 (3)	Br31—C32—C33—C38	2.7 (3)
C12—C13—C14—C15	0.0 (4)	C32—C33—C34—C35	0.0 (3)
C18—C13—C14—C15	178.2 (2)	C38—C33—C34—C35	179.0 (2)
C13—C14—C15—O11	179.2 (2)	C33—C34—C35—O31	179.8 (2)
C13—C14—C15—C16	−1.6 (4)	C33—C34—C35—C36	−0.7 (4)
O11—C15—C16—C11	−179.5 (2)	O31—C35—C36—C31	−179.9 (2)
C14—C15—C16—C11	1.3 (4)	C34—C35—C36—C31	0.6 (4)
C12—C11—C16—C15	0.6 (3)	C32—C31—C36—C35	0.2 (3)
C17—C11—C16—C15	−180.0 (2)	C37—C31—C36—C35	−178.9 (2)

### (4) 4-Bromo-3,5-dimethylphenol Hydrogen-bond geometry (Å, º)

**Table:**

*D*—H···*A*	*D*—H	H···*A*	*D*···*A*	*D*—H···*A*
O11—H11···O1	0.78 (3)	1.90 (3)	2.681 (3)	173 (4)
O21—H21···O11	0.76 (3)	1.92 (3)	2.682 (3)	176 (3)
O31—H31···O21	0.77 (3)	1.95 (3)	2.714 (2)	175 (3)
O1—H1···O31^i^	0.78 (3)	1.95 (3)	2.729 (3)	172 (3)

Symmetry code: (i) *x*−1/2, −*y*+3/2, −*z*+1.
